# Immune Cells Combined With NLRP3 Inflammasome Inhibitor Exert Better Antitumor Effect on Pancreatic Ductal Adenocarcinoma

**DOI:** 10.3389/fonc.2020.01378

**Published:** 2020-08-21

**Authors:** Hailiang Liu, Yong Xu, Kai Liang, Rong Liu

**Affiliations:** ^1^Department of Burn and Plastic Surgery, The Fourth Medical Center of Chinese PLA General Hospital, Beijing, China; ^2^The Second Hepatobiliary Surgical Department, The First Medical Center of Chinese PLA General Hospital, Beijing, China; ^3^General Surgery Institute, The First Medical Center of Chinese PLA General Hospital, Beijing, China

**Keywords:** pancreatic ductal adenocarcinoma, 3,4-methylenedioxy-β-nitrostyrene, cytokine-induced killer cells, NLRP3 inflammasome, immunotherapy

## Abstract

Pancreatic cancer is among the most aggressive malignancies associated with chronic inflammation. Moreover, cellular immunity can be inhibited by inflammation induced by nucleotide-binding domain, leucine-rich family, pyrin-containing 3 (NLRP3) inflammasome. Accordingly, NLRP3 inhibition combining cytokine-induced killer (CIK) cells may improve antitumor efficacy. 3,4-Methylenedioxy-β-nitrostyrene (MNS) was selected as a specific NLRP3 inflammasome inhibitor. Western blot was used to evaluate the NLRP3 inflammasome expression in pancreatic cancer cell lines SW1990 and PANC-1. The impact of NLRP3 inhibition on migration, invasiveness, and proliferation of pancreatic cancer cells was analyzed through wound healing assay, Transwell assay, and Cell Counting Kit-8 (CCK-8) assay, respectively. The combining antitumor effect *in vivo* of CIK and NLRP3 inhibition was evaluated in a subcutaneous human pancreatic cancer BALB/c nude mouse model. Western blot analysis showed significant expression of NLRP3 inflammasome in human pancreatic cancer lines SW1990 and PANC-1, and MNS did significantly inhibit the expression of NLRP3 inflammasome in cell lines. Moreover, NLRP3 inhibition could significantly decrease the migration, invasiveness, and proliferation of pancreatic cancer cells. *In vivo* experiments showed that combination treatment with MNS and CIK cells had the greatest antitumor effect among the four treatment groups including control, MNS, and CIK. Combination treatment with NLRP3 inflammasome inhibition and CIK cells showed greater antitumor efficacy through inflammation inhibition and immunity restoration.

## Introduction

Pancreatic ductal adenocarcinoma (PDAC) is one of the most prevalent and aggressive malignancies worldwide (1). In China, the morbidity and mortality of PDAC maintain rapidly increasing trend due to living standard improvement and diet structure changes, which contributed as the seventh most common cancer and the ninth leading cause of cancer-related death (2). Despite great advance in various conventional and emerging treatment approaches, surgery remains the only curative treatment approach for localized PDAC (3). However, ~80% of patients are inoperable due to tumor metastasis (4). Moreover, patients who underwent curative resection still suffer from high rates of perioperative morbidity and complication (5, 6). Other therapeutic options for PDAC are not very effective due to its resistance (7). Furthermore, conventional therapies may inhibit immune function and activate inflammation (8–11). And cancer patients present as aggressive inflammation and immunosuppression status (12). Thus, novel therapeutic strategies inhibiting tumor growth as well as restoring antitumor immunity and suppressing inflammation may exert a better antitumor effect.

It has been confirmed that inflammation plays an important role in development and therapeutic response of various malignancies (13). Epidemiological studies have shown that chronic pancreatitis is one of the major risk factors for PDAC, with a 2.3- to 18.5-fold increased risk compared to healthy controls, suggesting the development of PDAC is closely associated with inflammation (14, 15). Inflammasomes are intracellular multimolecular complexes that act as platforms for inflammation regulation (16). The nucleotide-binding domain, leucine-rich family, pyrin-containing 3 (NLRP3) inflammasome is currently the most characterized inflammasome, which consists of a scaffold protein (NLRP3), an apoptosis-associated speck-like protein containing a caspase-recruitment domain (ASC) adaptor, and caspase-1 (17). Activation of the NLRP3 inflammasome mediates the secretion of the pro-inflammatory cytokines interleukin (IL)-1β and IL-18 (18). Moreover, IL-1βcan enhance the invasive capacity of pancreatic cancer cells, while free IL-18 levels are increased in the blood of pancreatic cancer patients and are associated with poor survival (19, 20). Thus, inflammasome inhibition may suppress the pancreatic cancer cell growth via downregulation of IL-1β and IL-18.

Adoptive immunotherapy is an emerging means of cancer therapy, which has shown efficacy in several solid tumors including pancreatic cancer (21, 22). Immune cell-based cancer therapy eliminates cancer cells and restores antitumor immunity through the transfer of *ex vivo* expanded and activated immune cells. Currently, there are several immune cells applied in immunotherapy for cancer, such as dendritic cells, lymphokine-activated killer cells, natural killer cells, and cytokine-induced killer (CIK) cells (23, 24). CIK cells were generated from activation of human peripheral blood mononuclear cells (PBMCs) with IL-2, interferon (IFN)-γ, and anti-CD3 antibodies, inducing an enhanced cytotoxic effect (25). Moreover, CIK was characterized by aggressive antitumor activity and broad target tumor spectrum, which can proliferate rapidly *in vitro* and regulate immune environments (26). During the past several decades, CIK cell-based immunotherapy has shown antitumor efficacy in several malignancies (27, 28).

NLRP3 inflammasome in the tumor microenvironment inhibits antitumor T cell immunity by facilitating the migration of myeloid-derived suppressor cells (MDSCs) to the site of the tumor (29). Therefore, NLRP3 inflammasome inhibition can decrease pro-inflammatory cytokine secretion as well as eliminate antitumor T cell suppression. 3,4-Methylenedioxy-β-nitrostyrene (MNS), as a potent and specific inhibitor of the NLRP3 inflammasome, directly binds to NLRP3 and inhibits its ATPase activity in a concentration-dependent manner (30). In the present study, we demonstrate that MNS can induce suppression of proliferation, migration, and invasion of human pancreatic cancer cells through inhibiting NLRP3 inflammasome *in vitro* and *in vivo*, combining with CIK cells to exert a more aggressive antitumor effect. Our data provide the novel insight into combination treatment for PDAC through NLRP3 inflammasome inhibition and CIK infusion.

## Materials and Methods

### Cell Lines, Chemicals, and Reagents

The human PDAC cell line PANC-1 was obtained from the Tianjin Medical University Cancer Institute and Hospital (Tianjin, China). The human PDAC cell line SW1990 was obtained from general surgery institute in the Chinses PLA General Hospital (Beijing, China). PANC-1 was cultured in Dulbecco's modified Eagle's medium (DMEM; HyClone, USA) supplemented with 10% fetal bovine serum (FBS; HyClone, USA), 4 mM glutamine, 100 U/ml penicillin, and 100 μg/ml streptomycin. SW1990 was grown in RPMI 1640 medium with 10% FBS, 100 U/ml penicillin, and 100 μg/ml streptomycin, maintained in a humidified incubator at 37°C containing 5% CO_2_. Morphology and growth of each cell line were regularly monitored under microscopy. Cell viability was evaluated by staining with 0.4% trypan blue solution, while cell count was determined by using a hemocytometer. MNS was purchased from Sigma Aldrich (St. Louis, MO, USA), which was dissolved in 100% dimethyl sulfoxide (DMSO), finally forming a stock solution with a concentration of 20 mM and stored at −20°C and diluted with medium prior to use. The final DMSO concentration did not exceed 0.1% in each experiment. Anti-caspase-1, anti-IL-1β, anti-ASC, and anti-NLRP3 antibody were from Santa Cruz (Dallas, TX, USA).

### Cytokine-Induced Killer Cell Preparation and Evaluation

CIK cells were generated as previously described, with minor modifications (31). In brief, PBMCs at 5 × 10^6^ cells/ml were isolated from 26 healthy donors by Ficoll-Hypaque (Haoyang Biological Manufacture, Tianjin, China) gradient centrifugation. Then the PBMCs were resuspended at 5 × 10^6^ cells/ml in Cellix 601 media and stimulated with anti-CD3 (20 μg/ml) and anti-CD28 (1 μg/ml) antibody in the presence of recombinant human IFN-γ (1,000 U/ml) and recombinant human IL-2 (200 U/ml) for 5 days. The cell suspension then was furtherly cultured in Cellix 602 media containing recombinant human IL-2 (200 U/ml) for another 9 days. Fresh IL-2 and medium were replenished every 2–3 days. CIK cells finally harvested were stained with antibodies against CD3-PerCP/CD4-FITC/CD8-PE/CD56-APC, CD3- PerCP/CD8-PE/CD38-FITC/HLA-DR-PerC, and CD45RO-APC/CD62L-PE to evaluate the percentage of CD8+ T cells (CD3+CD8+), NK cells (CD3-CD56+), activated T cells (CD8+CD38+HLA-DR+), and central memory T cells (CD8+T_**CM**_, CD8+CD45RO+CD62L+) by a FACSCalibur flow cytometer (BD, United States).

### Western Blotting Analysis

Cells were washed twice with ice-cold phosphate buffered saline (PBS) and then harvested and lysed in cold radioimmunoprecipitation assay (RIPA) buffer. Equal amounts of protein were separated by 10% sodium dodecyl sulfate (SDS)-polyacrylamide gel electrophoresis (PAGE) and transferred to nitrocellulose membranes (Bio-Rad, Hercules, CA). The membrane was blocked with 5% nonfat milk and incubated with anti-NLRP3 antibody, ASC antibody, anti-caspase-1 antibody, and anti-IL-1β antibody (Santa Cruz Biotechnology, Inc., Dallas, TX, USA). Blots were developed using enhanced chemiluminescent substrate (Thermo Fischer Scientific Pierce, IL, USA).

### Monolayer Wound Healing Assay

Cells were seeded at density of 1 × 10^5^ cells/well in six-well-plates. After cells grown in six-well-plates had reached confluence, a scratch was made with a sterile 200-ml pipette tip. The medium and debris were aspirated away and replaced by fresh serum-free medium with DMSO or MNS, maintained in a humidified incubator at 37°C containing 5% CO_2_. The migration of the cells to the wound was monitored with an inverted Olympus phase-contrast microscope (Tokyo, Japan). Images were collected with a CCD camera at indicated time points to assess wound closure.

### Cell Migration and Invasion Assay

Cell migration and invasion were assessed by using 24-well transwell chambers according to manufacturer's instructions (BD Biosciences, USA). For the transwell migration assay, 5 × 10^4^ cells with or without MNS treatment were seeded on the top chamber of each insert (BD Biosciences, USA) with the noncoated polyethylene terephthalate (PET) membrane. For the invasion assay, 1 × 10^6^ cells with or without MNS treatment were placed on the upper chamber of each insert coated with Matrigel (BD Biosciences, MA). Then, 600 μl of medium supplemented with 30% FBS was injected into the lower chambers. After planned hours of incubation at 37°C, cells remaining in the top chambers or on the upper membrane of each insert were carefully removed with a cotton swab. Migrated cells adhering to the lower membrane of the inserts were fixed and stained with dye solution containing 0.1% crystal violet and 20% methanol, then counted and photographed through an inverted Olympus phase-contrast microscope (Tokyo, Japan).

### Cell Proliferation Assay

Cell proliferation was detected using the Cell Counting Kit-8 (CCK-8, Dojindo Molecular Technologies, Rockville, MD) assays in accordance with the manufacturer's instructions. Cells were placed into 96-well-plates at a density of 5 × 10^3^ cells/well and stabilized for 24 h at 37°C before being treated with MNS 0, 10, 20 μM. At 0, 12, 24, and 48 h after MNS treatment, 10 μl CCK-8 mixture solution was added to each well, and then the plates were incubated at 37°C in a 5% CO_2_ incubator for 2 h. The absorbance OD values were measured with a multiscan plate reader (Varioskan Flash, Thermo, CA, USA) at 450 nm.

### Xenograft Model and Treatments

The SW1990 cells (5 × 10^6^) were suspended in 100 μl serum-free RPMI 1640 and subcutaneously injected into the left upper flank of each mouse (female BALB/c-nu/nu, 4–6 weeks old). Two weeks after the cell injection, in the setting of observable tumors, all mice were randomly allocated to four groups (six per group), including (1) MNS group, (2) CIK group, (3) MNS+CIK combining treatment group, and (4) control group. Mice in the MNS group were only injected intraperitoneally with MNS (20 mg/kg body weight) every other day. CIK group received only intravenously 100 μl (1 × 10^6^ cells) CIK cells. Combining treatment group was administered intraperitoneally with MNS (20 mg/kg body weight) and intravenously with 100 μl (1 × 10^6^ cells) CIK cells, while the control group received 200 μl vehicle material. Tumor volumes were measured before each injection, which was calculated as described: V (cm^3^) = width^2^ (cm^2^) × length (cm)/2. Mice were treated every other day for 2 weeks. At the termination of the experiment, the mice were sacrificed by cervical dislocation, and the tumors were weighed immediately after dissection, then subjected to hematoxylin and eosin (H&E) staining and immunohistochemical analysis. Mice were manipulated and housed according to the recommendations in the Guide for the Care and Use of Laboratory Animals of the National Institutes of Health. The study was approved by the Committee on the Ethics of Animal Experiments of Chinese PLA General Hospital.

### Statistical Analysis

Analyses were performed with SPSS 20.0 (IBM, USA). *P* < 0.05 (two-sided) was considered statistically significant. Data were presented as means ± SEM, and comparisons were made using Student's *t*-test.

## Results

### Quantity and Quality of Cytokine-Induced Killer Cells

**Figure 1:**
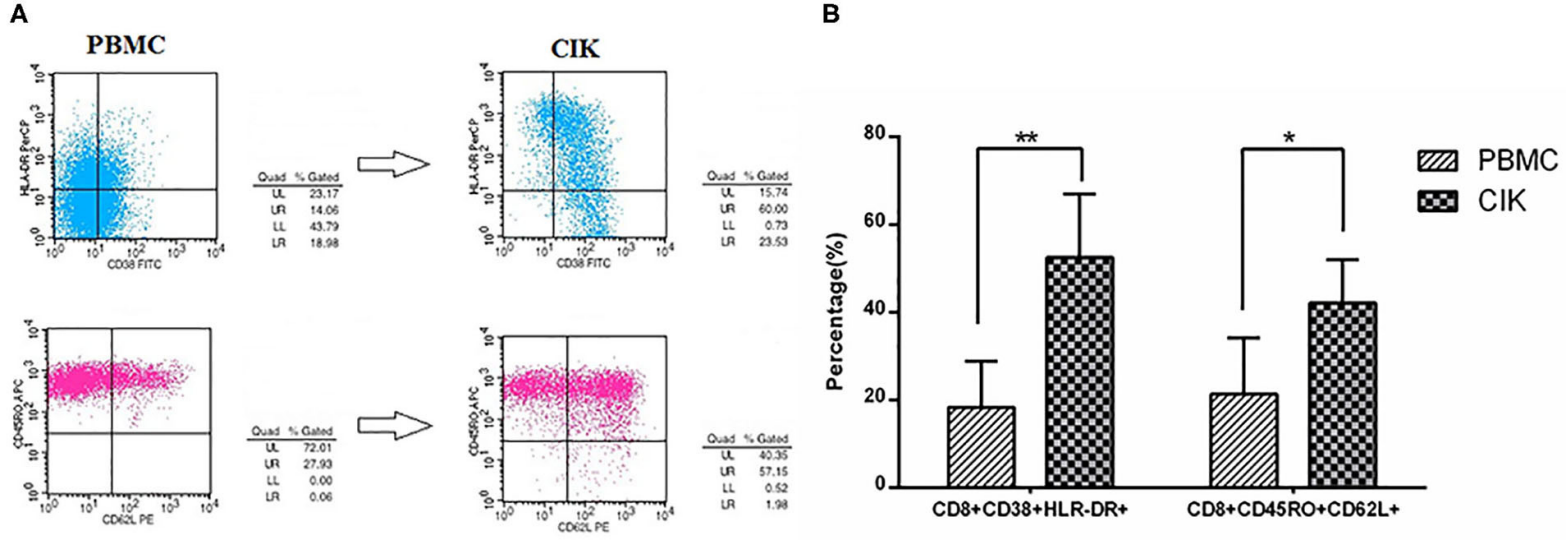
Prepared cytokine-induced killer (CIK) cells mainly consist of activated T cells and CD8+ T_CM_ by flow cytometry. **(A)** The plots of flow cytometry data of activated T cells and CD8+ T_**CM**_ in PBMCs and prepared CIK cells, respectively. **(B)** Compared with PBMCs, the percentage of CD8+CD38+HLA-DR+ cells was increased largely in CIK cells (*n* = 26, in CIK, 52.62 ± 13.53%; in PBMC, 18.35 ± 10.46%, **P < 0.01). Moreover, the percentage of CD8+CD45RO+CD62L+ cells in the CIK cells was increased to a high level after incubating for 12 days (*n* = 26, in CIK, 42.18 ± 9.87%; in PBMC, 21.37 ± 12.73%, *P < 0.05), indicating prepared CIK cells had been activated *in vitro* and had superior antitumor potential.

The mean prepared CIK cells count was 3.62 ± 1.23 × 10^8^. Moreover, the mean percentages of CD3+CD8+ T cells and CD3+CD56+ NKT cells in prepared CIK cells were 71.31 ± 18.77% and 34.62 ± 15.67%, respectively, suggesting CIK cells mainly consist of CD8+ T cells and NK cells. To evaluate the antitumor potential of CIK cells, we analyzed the proportion of activated T cells and central memory T cells in PBMCs and prepared CIK cells. The results showed that the mean percentage of activated T cells (52.62 ± 13.53% vs. 18.35 ± 10.46%, *P* < 0.01) and central memory T cells (42.18 ± 9.87% vs. 21.37 ± 12.73%, *P* < 0.05) in cultured CIK cells were dramatically higher than those of the PBMCs, respectively (Figures 1A,B).

### 3,4-Methylenedioxy-β-Nitrostyrene Downregulates the Expression Level of Nucleotide-Binding Domain, Leucine-Rich Family, and Pyrin-Containing 3 Inflammasome

**Figure 2:**
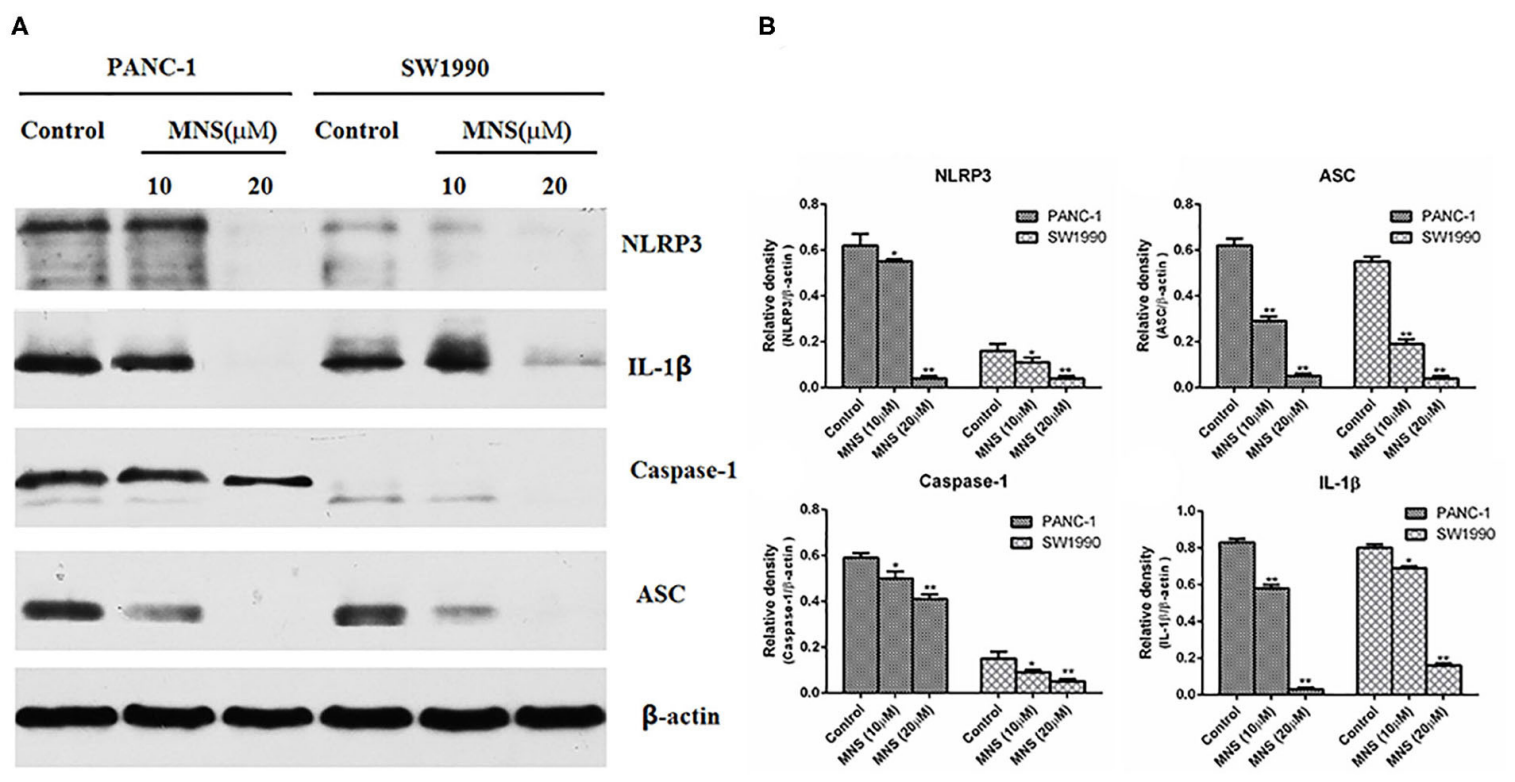
3,4-Methylenedioxy-β-nitrostyrene (MNS) downregulates the expression level of nucleotide-binding domain, leucine-rich family, and pyrin-containing 3 (NLRP3) inflammasome. **(A)** Western blot analyses of NLRP3 inflammasome and interleukin (IL)-1β protein expression in SW1990 and PANC-1 cells treated with two different concentrations of MNS for 12 h. **(B)** Relative quantitation of NLRP3 inflammasome and IL-1β. The results showed a significant decrease in the expression of NLRP3 inflammasome of PANC-1 and SW1990 cells treated with MNS (10 and 20 μM) compared with the non-treated control cells. *P < 0.05; **P < 0.01.

The NLRP3 inflammasome was considered as a positive regulator of tumor cell proliferation and metastasis. Verifying the expression of NLRP3 inflammasome on PDAC cells and furtherly confirming the inhibitory effect of MNS on the proliferation and metastasis of PDAC cells by the NLRP3 inflammasome downregulation. The expression levels of NLRP3 inflammasome of different treatment groups were determined. The results showed a significant decrease in the expression of NLRP3 inflammasome in PANC-1 and SW1990 cells treated with MNS (10 and 20 μM) compared with the non-treated control cells (Figures 2A,B). Furthermore, inflammasome mediates maturation and secretion of IL-1β and IL-18. The results showed that MNS decreased the expression level of IL-1β in both PANC-1 and SW1990 cells (Figures 2A,B), indicating that MNS suppresses the proliferation of PDAC cells by inhibition of NLRP3 inflammasome.

### 3,4-Methylenedioxy-β-Nitrostyrene Inhibits the Migration and Invasion of Pancreatic Ductal Adenocarcinoma Cells *in vitro*

**Figure 3:**
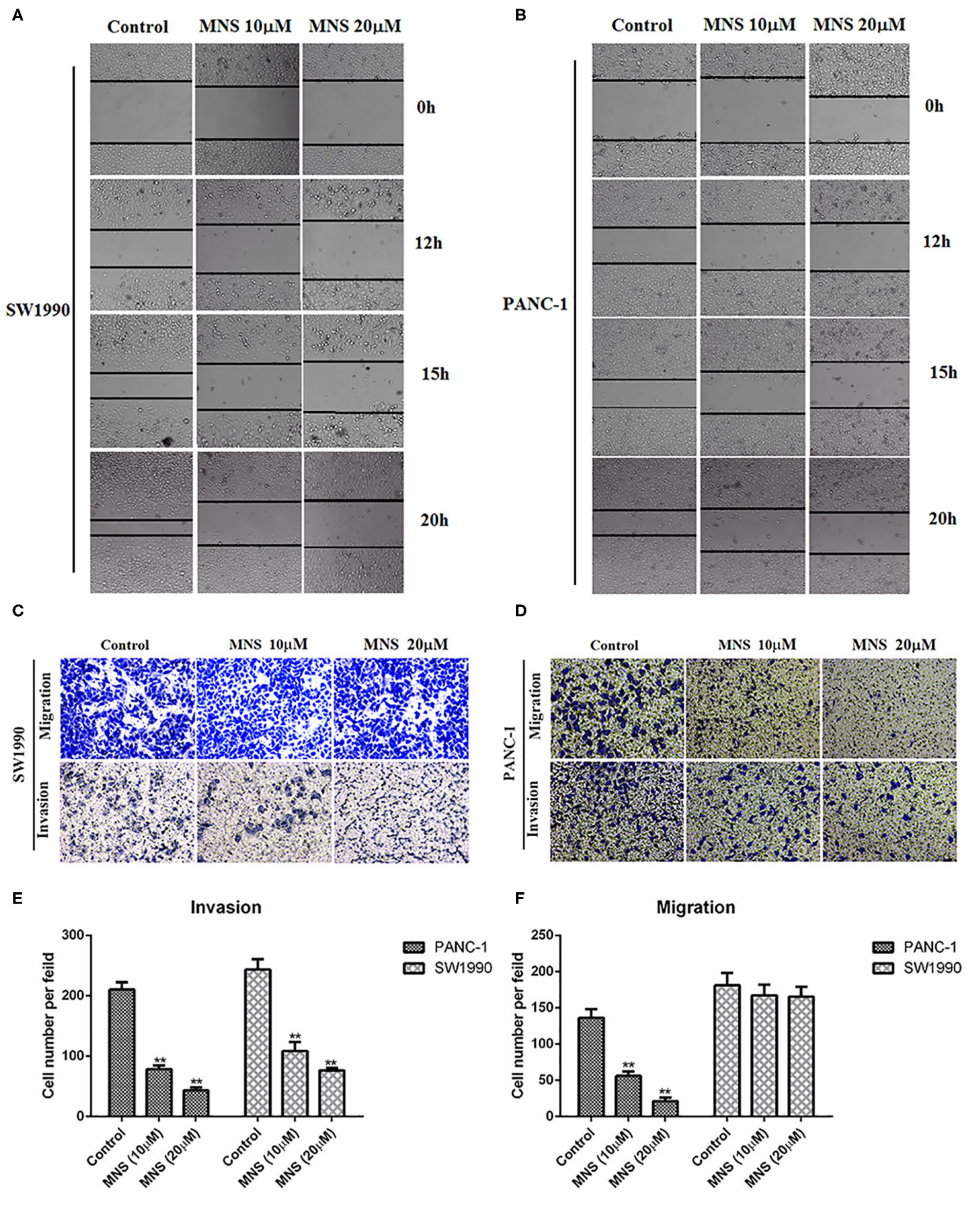
3,4-Methylenedioxy-β-nitrostyrene (MNS) inhibits the migration and invasion of pancreatic cancer cells. **(A,B)** The migration of cells into the wound was monitored in multiple wells. The images were acquired every hour for 20 h. The images shown represent 0, 12, 15, and 72 h. The distance between the two edges of the scratch in the MNS-treated cells was greater than that of the control. **(C,D)** MNS inhibits cell migration and invasion of PANC-1 and SW1990 in Transwell assay. Representative images are shown (×100 magnification). **(E,F)** Quantification of migration and invasion in Transwell assay. The values shown are expressed as the mean ± SEM. **P < 0.01 vs. non-MNS-treated control group.

Activation of NLRP3 inflammasome enhances the proliferation and migration of cancer cells. Thus, we analyzed whether MNS could affect the metastatic behaviors of cancer cells through NLRP3 inflammasome inhibition. In the wound healing assay, the wound in the control group was almost closed by migrated cells within 20 h, while cells treated with MNS either at 10 and 20 μM failed to migrate into and close a wound, as shown in Figures 3A,B. Moreover, we also determined the effect of MNS on cell migration of PANC-1 and SW1990 cells by Transwell migration assays and found that MNS treatment does not only inhibit SW1990 cells migration but also significantly inhibit PANC-1 cells in a concentration-dependent style (Figures 3C–F). The anti-invasion effect of MNS was analyzed through the Transwell invasion assay. MNS significantly inhibited PANC-1 and SW1990 cell invasion through a Matrigel-coated membrane in the chamber. In conclusion, MNS can inhibit both migration and invasion of human PDAC cells through NLRP3 inflammasome inhibition.

### 3,4-Methylenedioxy-β-Nitrostyrene Did Not Inhibit the Proliferation of Cytokine-Induced Killer Cells but Pancreatic Ductal Adenocarcinoma Cells *in vitro*

**Figure 4:**
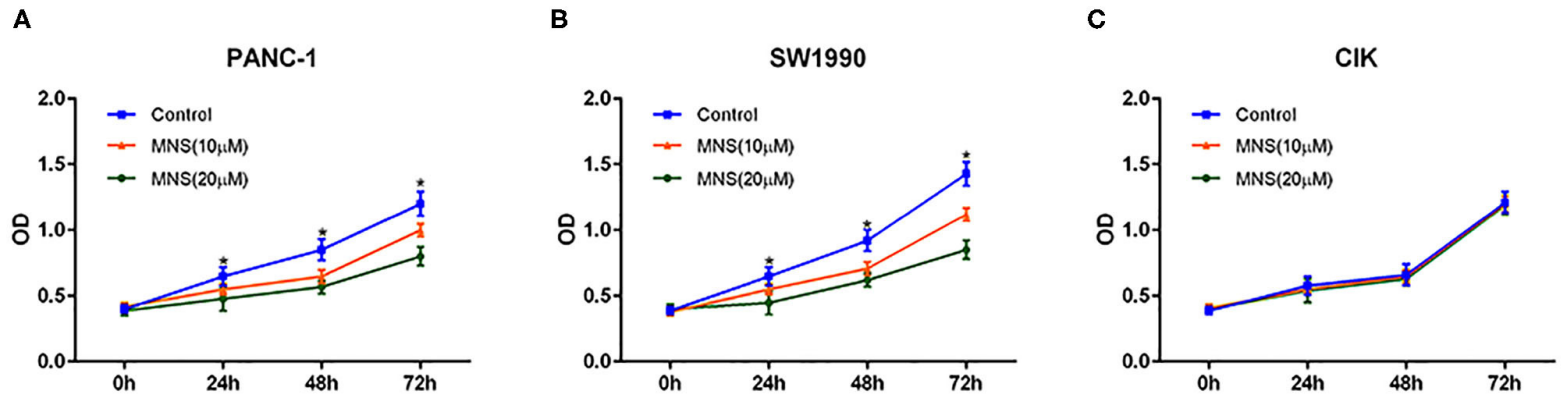
3,4-Methylenedioxy-β-nitrostyrene (MNS) inhibits the proliferation of pancreatic cancer cells. **(A,B)** Comparative dose- and time-dependent effect of MNS on the proliferation potential of SW1990 and PANC-1 cells. **(C)** There was no obvious effect of MNS on the proliferation potential of cytokine-induced killer (CIK) cells. The percentage of cell viability in the different treatment groups was determined using Cell Counting Kit-8 assay. *P < 0.05 vs. non-MNS-treated control group.

The effect of MNS on PDAC and CIK cell proliferation was analyzed by using CCK-8 assay. As shown in Figure 4, MNS significantly inhibited cell proliferation of PANC-1 and SW1990 in a dose- and time-dependent style (*P* < 0.05; Figures 4A,B). However, the proliferation of CIK cells did not become affected by MNS (Figure 4C). Such results suggested that NLRP3 inflammasome inhibition resulted because MNS cannot suppress the proliferation of CIK cells but PDAC cells.

### Antitumor Effect of Cytokine-Induced Killer Cells Combining 3,4-Methylenedioxy-β-Nitrostyrene on Pancreatic Ductal Adenocarcinoma *in vivo*

**Figure 5:**
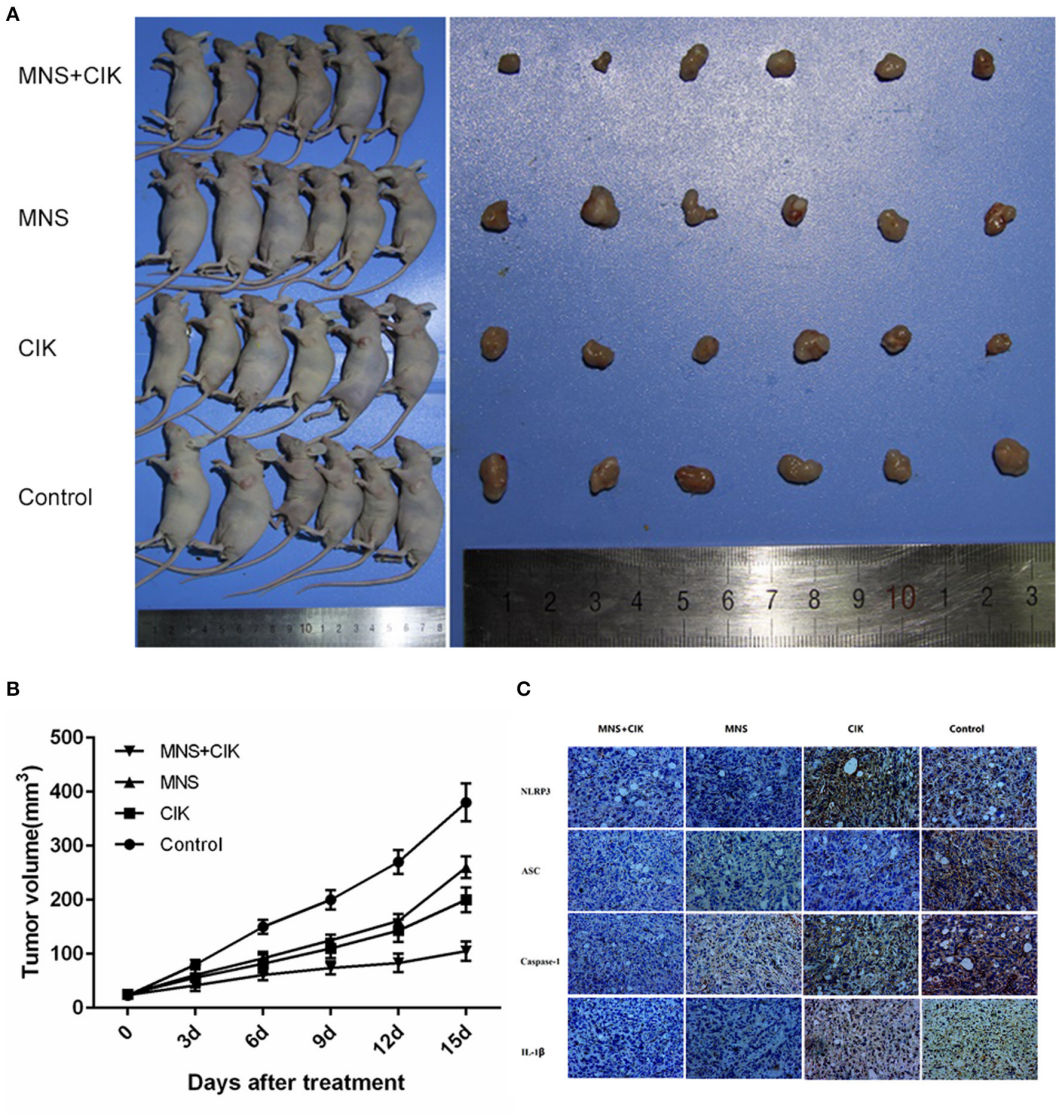
Cytkine-induced killer (CIK) cells combining 3,4-methylenedioxy-β-nitrostyrene (MNS) showed superior antitumor potential for pancreatic *in vivo*. **(A)** 1 × 10^6^ SW1990 cells was suspended in 100 μl serum-free RPMI 1640 and subcutaneously injected into the left upper flank of each mouse (female BALB/c-nu/nu, 4–6 weeks old). Two weeks after the cell injection, in the setting of observable tumors, all mice randomly allocated to the MNS group only received intraperitoneally MNS (20 mg/kg body weight) injection, CIK group only received intravenously 100 μl CIK cell injection, MNS+CIK combining treatment group was simultaneously treated with intraperitoneally MNS (20 mg/kg body weight) and intravenously 100 μl CIK cells, and the control group received 200 μl vehicle. **(B)** Tumor volumes were measured before each injection, which was calculated as described: *V* (cm^3^) = width^2^ (cm^2^) × length (cm)/2. Tumor growth curves showed that the average volume of tumors in the MNS+CIK group was significantly smaller compared to the control group and both single-treatment groups (*P* < 0.05). **(C)** Representative immunohistochemical analysis of tumor samples showed that expression of NLRP3 inflammasome and interleukin (IL)-1β were inhibited in the MNS+CIK group.

The results obtained from *in vitro* studies revealed that MNS did not inhibit the proliferation, migration, and invasion capacity of CIK cells but PDAC cells through downregulating NLRP3 inflammasome. To furtherly evaluate the *in vivo* antitumor effects of CIK combining with MNS, we performed an *in vivo* proliferation study. Tumor growth curves showed that the average volume of tumors in the MNS+CIK group was significantly smaller compared to those of the control group and both single-treatment groups (*P* < 0.05; Figures 5A,B). The mean tumor weight of MNS+CIK group was also significantly lower than those of the control group and both single-treatment groups, with tumor growth inhibition rate of 72.33% (Table 1). Moreover, no animals died during the treatment, and all of them grew well without dyspraxia. There was no significant difference in body weight among the four groups before and after treatment.

**Table 1:** Comparison of tumor weight and inhibition rate among different treatments.

	**Tumor weight (g)**	**Inhibition rate (%)**
Control	1.522 ± 0.240	
MNS	0.965 ± 0.210^*^	36.60
CIK	0.632 ± 0.100^*^	58.48
CIK + MNS	0.421 ± 0.110^*^	72.33

* *P < 0.05. CIK, cytokine-induced killer; MNS, 3,4-methylenedioxy-β-nitrostyrene*.

Immunohistochemical analysis of tumor samples showed that the expressions of NLRP3 inflammasome and IL-1β were inhibited in MNS+CIK groups (Figure 5C), furtherly indicating that MNS inhibits tumor growth through NLRP3 inflammasome inhibition.

Taken together, MNS combining CIK cells had more aggressive antitumor potential for PDAC through NLRP3 inflammasome inhibition and immunity restoration.

## Discussion

PDAC is characterized by the most aggressive malignancies and poor prognosis. Conventional managements including surgery, chemotherapy, and radiotherapy can kill tumor cells, which also simultaneously cause inflammation response and immunosuppression facilitating the relapse and metastatic potential (8). Moreover, cancer patients themselves present as aggressive inflammation and immunosuppression status (32). Furthermore, epidemiological studies and molecular biology have revealed that the development of pancreatic cancer is associated with chronic inflammation (15). This emphasizes the need for a novel treatment mode for pancreatic cancer by combining inflammation regulation and immunity restoration.

The role of excessive inflammatory response in contributing to cancer progression and metastasis has been well-documented (33, 34). Previous studies have emphasized that pro-inflammatory cytokines facilitate pro-carcinogenic activity by triggering the secretion of vascular endothelial growth factor (VEGF), fibroblast growth factor 2 (FGF2), and signal tranducer and activator of transcription 3 (STAT3) and subsequently support cancer survival and distant metastasis, particularly IL-1β and IL-18 (35–37). Furthermore, IL-1β can enhance the invasive capacity of pancreatic cancer cells, while free IL-18 levels are increased in the blood of pancreatic cancer patients and are associated with poor survival (19, 20). Therefore, inhibiting the molecular network of inflammasomes may become a novel strategy for cancer prevention research.

The inflammasome is a multiprotein complex that acts as a platform for host immune activation and inflammatory response to dangerous stimuli (38). In the setting of activation *via* recognizing danger signals, the inflammasome recruits and activates caspase-1, thus inducing the precursors of IL-1β and IL-18 to their mature forms (39). The NLRP3 inflammasome, one of the best characterized inflammasomes, consisting of NLRP3 protein, ASC adapter protein, and pro-caspase-1, potently modulates the innate immune function and inflammatory response by regulating the maturation of pro-inflammatory cytokines IL-1β and IL-18. It has been shown that NLRP3 inflammasome plays an important role in the development of many cancer types (40, 41). However, Whether NLRP3 inflammasome contributes to proliferation and metastasis of pancreatic cancer is still unknown. Therefore, in this study, we evaluated the expression of NLRP3 inflammasome on pancreatic cancer cells and showed that each component of NLRP3 inflammasome was expressed on both human pancreatic cell lines SW1990 and PANC-1, confirming the relationship between NLRP3 inflammasome and pancreatic cancer.

To furtherly analyze the role of the NLRP3 inflammasome in pancreatic cancer progression, we selected a novel specific NLRP3 inflammasome inhibitor, MNS (42). MNS, a Syk kinase inhibitor, which inhibits NLRP3 inflammasome activity directly binds to NLRP3 and inhibits its ATPase activity in a concentration-dependent manner (30). Our results showed that MNS could significantly decrease the expression of NLRP3 inflammasome and IL-1β of SW1990 and PANC-1 cells. Furthermore, MNS also significantly inhibited the migration, invasion, and proliferation of pancreatic cancer cells *in vitro*. Based on the results of the present study, we confirmed that MNS inhibits pancreatic cancer cell growth through suppressing NLRP3 inflammasome.

*Ex vivo* activation of human PBMCs through IL-2 and anti-CD3 antibodies is known to potently generate the cytotoxic effector CIK cells (43). In the present study, we prepared CIK cells from human PBMCs with two activators IL-2 and anti-CD3 and anti-CD28 antibody. And we showed that CIK cells mainly consisted of activated T cells and memory T cells, which not only survive longer but also have superior antitumor potential and eradicate large established tumors (44). However, antitumor immunity of T cells was inhibited by NLRP3 inflammasome in the tumor microenvironment.

In this study, the results of CCK-8 assay show that MNS can inhibit the proliferation of SW1990 and PANC-1 cells but had no obvious influence on *ex vivo* prepared CIK cells, indicating that simultaneous application of NLRP3 inflammasome inhibition and CIK cell infusion is preferable for cancer treatment. Therefore, we evaluated the antitumor effect of CIK cells combining with MNS in a pancreatic cancer xenograft model and found that combination treatment had better antitumor effect than single treatment but had no influence on mouse growth, furtherly confirming that combination treatment with NLRP3 inflammasome inhibition and CIK cell infusion shows greater antitumor efficacy through inhibition of cancer-related inflammation and restoration and promotion of antitumor immunity.

## Conclusions

This study firstly showed that the proliferation, invasion, and metastasis of pancreatic cells were suppressed through inhibition of NLRP3 inflammasome. Moreover, we firstly confirmed that combining inhibition of cancer-related inflammation and antitumor restoration had a superior antitumor potential. Our findings provide an important basis for a novel adjuvant therapy model of pancreatic cancer.

## Data Availability Statement

All data analyzed and generated during the current study are available from the corresponding author upon reasonable request.

## Ethics Statement

The animal study was reviewed and approved by Ethics of Animal Experiments of Chines PLA General Hospital.

## Author Contributions

HL, YX, and RL designed all the experiments. HL and KL conducted the experiments and wrote the manuscript. YX assisted in all animal experiments and wound healing assay, Transwell assay, and CCK-8 assay. All authors read and approved the final manuscript.

## Conflict of Interest

The authors declare that the research was conducted in the absence of any commercial or financial relationships that could be construed as a potential conflict of interest.
