# Corrigendum: Immune Cells Combined With NLRP3 Inflammasome Inhibitor Exert Better Antitumor Effect on Pancreatic Ductal Adenocarcinoma

**DOI:** 10.3389/fonc.2021.817747

**Published:** 2021-12-22

**Authors:** Hailiang Liu, Yong Xu, Kai Liang, Rong Liu

**Affiliations:** ^1^ Department of Burn and Plastic Surgery, The Fourth Medical Center of Chinese PLA General Hospital, Beijing, China; ^2^ The Second Hepatobiliary Surgical Department, The First Medical Center of Chinese PLA General Hospital, Beijing, China; ^3^ General Surgery Institute, The First Medical Center of Chinese PLA General Hospital, Beijing, China

**Keywords:** pancreatic ductal adenocarcinoma, 3,4-methylenedioxy-b-nitrostyrene, cytokine-induced killer cells, NLRP3 inflammasome, immunotherapy

In the original article, there was a mistake in the legend for **Figure 1** as published. The stated DC-AT was a mistake. The correct legend appears below.

**Figure 1:**
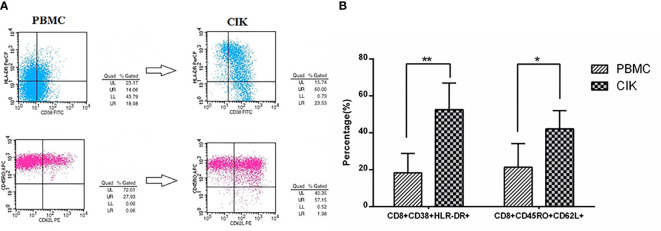
Prepared cytokine-induced killer (CIK) cells mainly consist of activated T cells and CD8+Tcm by flow cytometry. **(A)** The plots of flow cytometry data of activated T cells and CD8+Tcm in PBMCs and prepared CIK cells, respectively. **(B)** Compared with PBMCs, the percentage of CD8+CD38+HLA-DR+ cells was increased largely in CIK cells (n = 26, in CIK, 52.62 ± 13.53%; in PBMC, 18.35 ± 10.46%, **P < 0.01). Moreover, the percentage of CD8+CD45RO+CD62L+ cells in the CIK cells was increased to a high level after incubating for 12 days (n = 26, in CIK, 42.18 ± 9.87%; in PBMC, 21.37 ± 12.73%, *P < 0.05), indicating prepared CIK cells had been activated *in vitro* and had superior antitumor potential.

In the original article, there was a mistake in **Figure 5C** as published. Another figure was mistakenly used when arranging **Figure 5C**. The corrected **Figure 5** appears below.

**Figure 5:**
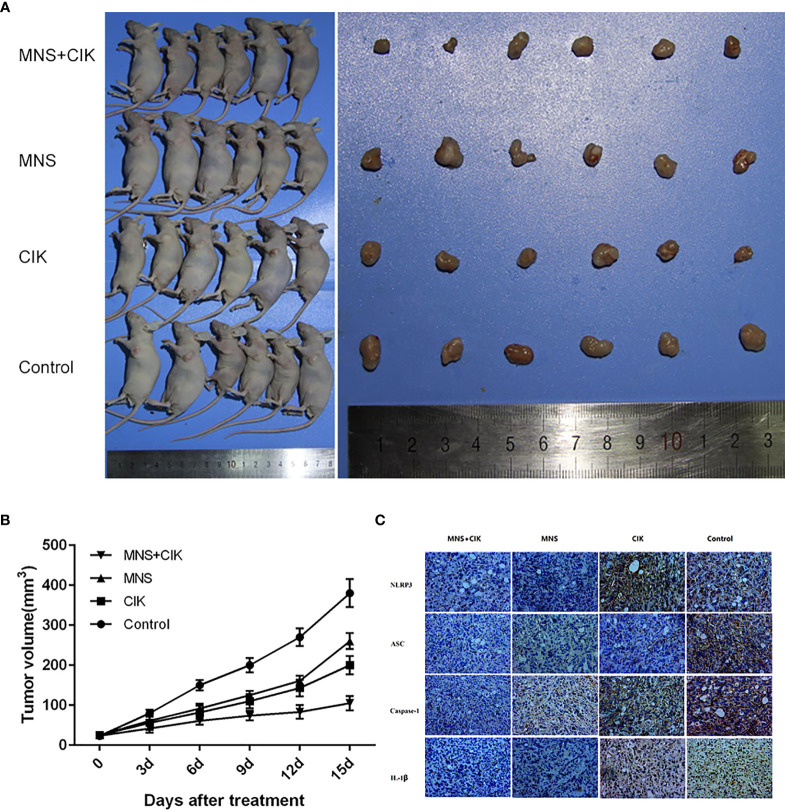
Cytokine-induced killer (CIK) cells combined with 3,4-methylenedioxy-β-nitrostyrene (MNS) showed superior antitumor potential for pancreatic cancer *in vivo*. **(A)** 1 ×10^6^ SW1990 cells were suspended in 100 μl serum-free RPMI 1640 and subcutaneously injected into the left upper flank of each mouse (female BALB/c-nu/nu, 4–6 weeks old). Two weeks after the cell injection, in the setting of observable tumors, mice were randomly allocated to the MNS group, which only received an MNS (20 mg/kg body weight) injection intraperitoneally, the CIK group, which only received a 100 μl CIK cell injection intravenously, the MNS+CIK combined treatment group which was simultaneously treated with intraperitoneal MNS (20 mg/kg body weight) and intravenous CIK cells (100 μl), and the control group which received 200 μl of vehicle. **(B)** Tumor volumes were measured before each injection, which were calculated as described: V (cm3) = width2 (cm^2^) × length (cm)/2. Tumor growth curves showed that the average volume of tumors in the MNS+CIK group was significantly smaller compared to the control group and both single-treatment groups (P < 0.05). **(C)** Representative immunohistochemical analysis of tumor samples showed that expression of NLRP3 inflammasome and interleukin (IL)-1β were inhibited in the MNS+CIK group.

The authors apologize for this error and state that this does not change the scientific conclusions of the article in any way. The original article has been updated.

## Publisher’s Note

All claims expressed in this article are solely those of the authors and do not necessarily represent those of their affiliated organizations, or those of the publisher, the editors and the reviewers. Any product that may be evaluated in this article, or claim that may be made by its manufacturer, is not guaranteed or endorsed by the publisher.

